# Virtual restorative environment therapy as an adjunct to pain control during burn dressing changes: study protocol for a randomised controlled trial

**DOI:** 10.1186/s13063-015-0878-8

**Published:** 2015-08-05

**Authors:** Charlotte Small, Robert Stone, Jane Pilsbury, Michael Bowden, Julian Bion

**Affiliations:** School of Clinical and Experimental Medicine, The Medical School, Vincent Drive, University of Birmingham, Birmingham, B15 2TT UK; School of Electronic, Electrical and Systems Engineering, University of Birmingham, Edgbaston, Birmingham, B15 2TT UK; Anaesthetic Department, Queen Elizabeth Hospital Birmingham, Mindelsohn Way, Edgbaston, B15 2WB UK; The Medical School, Vincent Drive, University of Birmingham, Birmingham, B15 2TT UK

**Keywords:** procedure-related pain, distraction, virtual reality, burns, pilot study, randomised controlled trial

## Abstract

**Background:**

The pain of a severe burn injury is often characterised by intense background pain, coupled with severe exacerbations associated with essential procedures such as dressing changes. The experience of pain is affected by patients’ psychological state and can be enhanced by the anxiety, fear and distress caused by environmental and visual inputs. Virtual Reality (VR) distraction has been used with success in areas such as burns, paediatrics and oncology. The underlying principle of VR is that attention is diverted from the painful stimulus by the use of engaging, dynamic 3D visual content and associated auditory stimuli. Functional magnetic resonance imaging (fMRI) studies undertaken during VR distraction from experimental pain have demonstrated enhancement of the descending cortical pain-control system.

**Methods/Design:**

The present study will evaluate the feasibility of introducing a novel VR system to the Burns Unit at the Queen Elizabeth Hospital Birmingham for dressing changes: virtual restorative environment therapy (VRET). The study will also explore the system’s impact on pain during and after the dressing changes compared to conventional analgesia for ward-based burn dressing changes. A within-subject crossover design will be used to compare the following three conditions:

1. Interactive VRET plus conventional analgesics.
2. Passive VRET with conventional analgesics.
3. Conventional analgesics alone.

Using the Monte Carlo method, and on the basis of previous local audit data, a sample size of 25 will detect a clinically significant 33 % reduction in worst pain scores experienced during dressing changes.

**Discussion:**

The study accrual rate is currently slower than predicted by previous audits of admission data. A review of the screening log has found that recruitment has been limited by the nature of burn care, the ability of burn inpatients to provide informed consent and the ability of patients to use the VR equipment. Prior to the introduction of novel interactive technologies for patient use, the characteristics and capabilities of the target population needs to be evaluated, to ensure that the interface devices and simulations are usable.

**Trial registration:**

Current Controlled Trials ISRCTN23330756. Date of Registration 25 February 2014.

## Background

The management of severe burns involves meticulous wound care in order to prevent infection. This necessitates regular debridement and dressing changes over a period of days to weeks. This form of wound care is recognised as one of the most painful procedures that can be undertaken, with some patients reporting severe to excruciating pain [1]. Poor acute pain management can have long-lasting consequences and is associated with adverse physical and psychological sequelae such as persistent pain, depression, post-traumatic stress disorder and, at the extreme, suicidal ideation [2–4]. Initial dressing changes and wound care requiring skin grafting are usually carried out under general anaesthesia. Subsequent wound care may be undertaken on a regular basis, often daily, with the benefits of general anaesthesia exceeded by the risks of anaesthesia and the practical constraints of operating theatre access. Currently, sedoanalgesia or opioid-based analgesia, in addition to other agents such as paracetamol, is used for pain relief during ward-based wound care [5], but these agents have multiple unpleasant side effects. Some units use distraction techniques informally or as part of a multi-modal approach to pain relief. These range from simple interventions such as video watching or listening to music, to more high tech interventions such as virtual reality (VR)-based computer systems [3].

Computer-generated, three-dimensional ‘virtual’ worlds have existed in the fields of gaming, education and simulation for nearly three decades [6] with applications in the medical domain only reaching an acceptable level of maturity in the past 5 to 10 years or so [7, 8]. Computer hardware, such as motion-tracking, head mounted displays and noise-cancelling headphones, allows ‘immersion’ in, and interaction with, a virtual environment. Pain is enhanced by the anxiety, fear and distress caused by environmental and visual inputs. The underlying principle of VR is that attention is diverted from the painful stimulus through the use of engaging, dynamic content presented (primarily) visually and aurally, with some systems capable of delivering force and touch (haptic) sensations and limited olfactory stimulation. Functional magnetic resonance imaging (fMRI) studies undertaken during VR distraction from experimental pain have demonstrated enhancement of the descending cortical pain-control system via activation of the perigenual anterior cingulate cortex and periaqueductal grey matter [9]. A number of studies have investigated the use of VR during painful burn management and physiotherapy, showing a statistically and clinically significant reduction in pain scores of around 30 % [10–12]. It has been found to be especially effective for those experiencing severe pain [10]. Preliminary studies also suggest that the effectiveness of VR does not decline with multiple treatments, with studies showing it retained its analgesic potency over multiple sessions [13, 14].

Viewing natural environments has been shown to have therapeutic benefits, including improved pain relief and post-operative recovery [15]. Indeed, static images have recently been introduced to the Burns Unit at the Queen Elizabeth Hospital, Birmingham (QEHB). Many of these, however, are not visible to patients within their rooms. A view of nature could be provided as a virtual world - bringing the restorative natural environment to the patient’s bedside. No system to date has integrated a virtual restorative environment with distraction therapy [16–18].

Acceptance of interactive technologies, including virtual reality, by the users (patients and staff) is dependent on usability (in some cases, wearability), utility and affordability. Developments driven by the gaming industry have resulted in rapid advancements in affordability, portability and ubiquity of computer systems [19, 20]. These advances present great opportunities to develop and evaluate a novel VR system for pain management at the patient bedside, using commercially available off-the-shelf equipment. Low-cost devices have been developed by other users, but use commercial video games, designed for use by children [21].

Using an active human-centred approach to prototype development and considering context, ergonomics and task completion increases the usability and utility of the intervention [22].

Efficacy of our proposed system is based on previous work on VR distraction [23, 24]. Uniquely, our proposed system will integrate distraction therapy with the development of virtual restorative environments that are designed to appeal to adults. We have termed our system ‘Virtual Restorative Environment Therapy’ (VRET).

### Context

The environmental context of burn patients and their carers at QEHB is a teaching hospital with a specialist burns unit. Most patients are in generously proportioned single or double rooms, but most lack windows with a view and natural light. The prototype system must be mobile and easily moved between patient bed spaces, with appropriate decontamination procedures to minimize the risk of cross-infection.

### Ergonomics

The pattern of burn injury will vary between every patient. As patients may have restrictions to movement and hand or arm function, interface devices, such as commercial, off-the-shelf (COTS) hand controllers and gamepads, can be chosen or modified to allow optimum use by those with the greatest functional deficits. Some analgesics, for example opiates and antidepressants, may affect perception and coordination [25]. These factors have been considered in the development of the VR system, particularly with regard to the fidelity of the images presented and the mapping of the functions of the control devices used onto the computer-generated objects displayed on screen [26].

### Task

A priority of the prototype design is to ensure that the process of changing the dressing is in no way hindered. The layout of the patient, bed, equipment trolley and nursing staff will vary depending on the location of the burn wounds. The VRET prototype has been configured to allow flexibility in screen location and patient access. Free-flowing water is also used for some dressing changes to enhance tissue and dressing removal. Full immersion in water, or the use of a ‘hydrotank’ is not used during dressing changes at QEHB, removing the requirement for a fully waterproof system [27].

### Hypothesis

When compared to standard analgesia alone, adjunctive immersive interaction with a virtual environment will reduce pain during ward-based burn dressing changes.

### Aims

To assess the efficacy of the immersive virtual reality system in terms of the following:

1. The effects on the pain experienced by patients during painful procedures requiring multimodal analgesia but not requiring sedation/anaesthesia.
2. The effects on the anxiety experienced by patients during painful procedures not requiring sedation/anaesthesia.
3. The system’s ability to generate a sense of presence on the part of the VR users^1^
4. User acceptance experienced by the patients and usability feedback.
5. User acceptance by the clinical staff caring for the patients.

## Methods/Design

This study will be a single-centre block-randomised cross-over trial.

### Ethics

This study has been approved by the National Research Ethics Service Committee South Birmingham (Reference 13/WM/0205) and registered with the UK Clinical Research Network portfolio (Study ID 15785) and Current Controlled Trials (ISRCTN23330756).

### Intervention

**Fig. 1:**
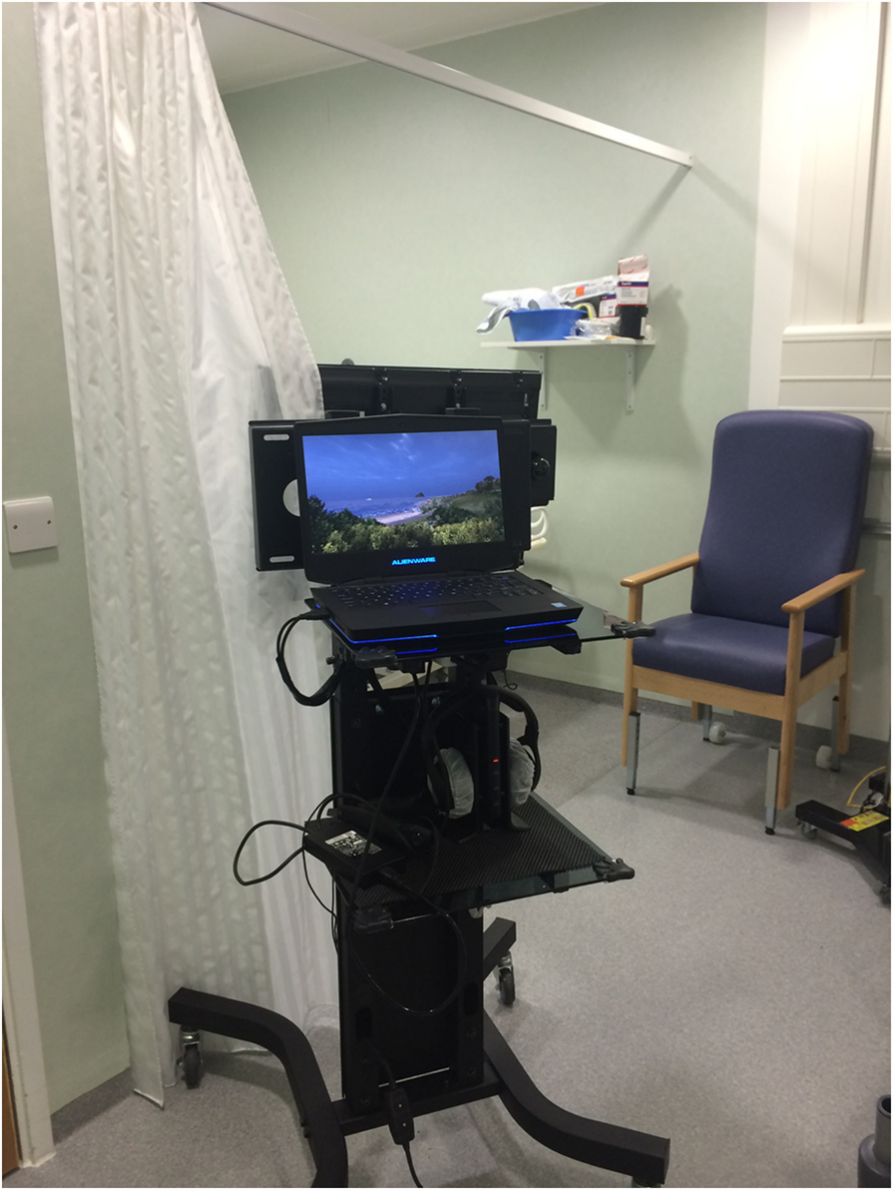
Virtual restorative environment therapy (VRET) system *in situ*, viewed from the rear

**Fig. 2:**
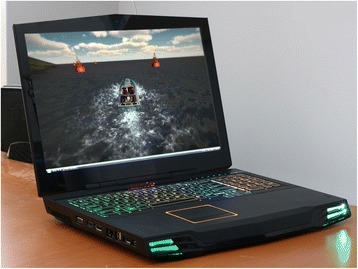
Screenshot of the speed boat game viewed on laptop

The prototype has been constructed using low-cost, commercial, off-the-shelf components (Fig. 1). Wound care is carried out in the patient bed space; thus, each prototype is fully mobile and can be placed at the end or side of each bed on the burns unit. The prototype consists of a high definition, 32-inch screen, headphones and hand controller (chosen for single-hand use and equipped with a thumb-operated joystick). The use of a head-mounted display [28] was considered but deemed to be unacceptable due to potential discomfort [29], hygiene issues and the inability of patients with facial or scalp burns to use such a device. The authors are aware that previous research has suggested that the use of head mounted displays may improve immersion and presence (Box 1) [30] in the virtual world. Thus, the study design includes the assessment of immersion for each patient following the VRET interventions. During passive video VRET, participants look at a static image of a virtual seascape. In the active VRET treatment participants are able to navigate the virtual world, traveling in a speed boat (Fig. 2). The activity will have multiple sensory inputs, encouraging maximum attention, yet be simple enough to be undertaken by those with impairment due to physical limitations and performance limitations such as opiates, pain and sleep deprivation.

### Study participants

#### Inclusion criteria

Inclusion criteria are patients with burns (any cause) admitted to the Queen Elizabeth Hospital Burns Unit requiring the following:

1. at least three in-patient dressing changes.
2. opioid-based analgesia (for example, oral morphine, codeine phosphate or tramadol) or inhaled nitrous oxide (entonox) for the dressing change (that is, patients who may potentially experience moderate or severe pain).

#### Exclusion criteria

Exclusion criteria include the following:

1. Inability to use the VRET equipment (for example, blindness or severe bilateral hand injuries).
2. Requirement for general anaesthesia or sedation with ketamine or midazolam.
3. Poor cognitive state (for example, severe dementia, delirium or severe psychiatric illness).
4. Multidrug-resistant infection (due to potential equipment contamination, although low risk, this criterion is appropriate for the feasibility study).
5. Dressing changes requiring overhead showering

### Randomisation process

The order of intervention will be randomised using block allocation, as is appropriate for the small sample size [31]. A computer-generated randomisation sequence will use a block size of six, which will be repeated four times.

### Sample size

Using a Monte Carlo method, we estimate that a sample size of 25 patients would provide 99 % power to detect a clinically important effect. The data and assumptions underlying the calculation are as follows:

1. The observed distribution of 33 pain scores in a recent audit is the true distribution of the ‘control’ value.
2. The mean percentage reduction from the control value is 30 for interactive VRET. This value has been chosen as it has been demonstrated that the patient-determined, clinically important improvement reduction in pain experienced corresponds to 33 % reduction in measured pain, using an 11-point numerical rating scale [32].
3. The mean percentage reduction from the control value is 15 for passive VRET.
4. Both percentage reductions are normally distributed with a standard deviation of 25.
5. The nonparametric Friedman test (with a significance level of 5 %) will be used to compare the pain scores under the three conditions.

### Procedure

Patients will be informed of the study by their clinical team. Patients meeting the inclusion criteria will be invited to participate by the research team, following which they will have 12 to 24 h to decide whether they wish to enrol in the study, at which point written informed consent will be taken.

Each patient will receive each condition; active VRET, passive VRET and control. The order will be randomised prior to the first procedure.

Analgesia will be provided as per ward protocol for each intervention and patient requirement prior to dressing change. Dressing changes will be carried out as usual by ward staff. Regular analgesics will be given at set times according to prescription (for example, 07:00, 12:00, 14:00, 17:00, 22:00) and, as required, analgesia will be given 30 to 60 min prior to a dressing change. These timings will be recorded. Breakthrough pain is managed by nurse-titrated boluses of intravenous morphine.

For conditions utilising the VRET equipment, a member of the research team will set up the equipment in the patient room/bed space and patients will receive a 5 to 10 min tutorial/demonstration prior to their dressing change. The setup procedures for the VRET system will be demonstrated to the nurse undertaking the dressing change to allow evaluation of nursing perception of the system, including ease of use.

A member of the research team will remain in the room whilst the dressing change is undertaken by a member of the burn unit’s nursing team in order to detect adverse events or troubleshoot where required. Their intervention will be the minimum possible in order to allow normal clinical proceedings. Following the dressing change, a member of the research team will remove the equipment from the patient’s bed space/room, decontaminate it and store it safely.

A researcher, who has been blinded to the intervention, will return one hour following the dressing change, to collect the questionnaire data from the patient on their experience of pain, anxiety and nausea during their dressing change. Despite the disadvantage of relying on recalled pain by the patient, asking patients questions during the distraction activity could impact the efficacy of the analgesic intervention. Likewise, asking questions during the control condition could be distracting in itself. This methodology will be repeated for the two subsequent dressing changes. At the end of the study period, patients will be offered the use of the VRET system for future dressing changes if they found it beneficial.

The member of the research team present for the dressing change will then undertake a semi-structured interview and questionnaire of the patient and nursing staff to assess their user acceptance of the prototype VRET system.

### Outcome measures

#### Primary

The primary outcome measure is the worst pain score experienced during burn dressing change (11-point numerical rating scale - NRS). The NRS has been selected as the patient does not have to be able to write, as is required for a graphical rating scale.

#### Secondary

The secondary outcome measures are as follows:

1. Average pain scores during dressing change (NRS).
2. Pain score 1 h after dressing change (NRS).
3. Anxiety score during dressing change (NRS).
4. Patient satisfaction with pain control, measured by Likert item in response to the following question:
  - How satisfied were you with your overall pain management during your dressing change?
5. Patient usability, measured by Likert items in response to the following questions:
  - To what extent did you feel the VR equipment was easy to use?
  - Have you enjoyed the VR session?
  - To what extent did you feel you ‘went into’ the virtual world?
  - How satisfied were you with the virtual reality system during your dressing change?
6. Nausea experienced during the dressing change (Likert item).
7. Nursing satisfaction with interactive distraction system, measured by Likert items in response to the questions:
  - To what extent did you feel the VR equipment was easy to use?
  - To what extent did you feel the VR equipment interfered with clinical care?

All but the nausea assessment has five possible responses: not at all, not really, undecided, somewhat and very much. The nausea Likert item consists of not at all, very briefly, some of the time, most of the time and all of the time. Each Likert item will be analysed individually.

Demographic data will include patient age, gender, size of burn, time since injury and pain score before the dressing change.

### Data analysis

Data will be analysed using IBM SPSS 20.0. Normality will be assessed using the Shapiro-Wilk Test. When comparing the three conditions the repeated measures analysis of variance (ANOVA), assuming normality, or the Friedman test will be used. When comparing passive or active VRET conditions to the control condition, the Wilcoxon signed rank test will be a possible alternative to a paired *t* test.

## Discussion

The study accrual rate is currently slower than predicted by previous audits of admission data. A review of the screening log has found that recruitment has been limited by the nature of burn care, the ability of burn inpatients to provide informed consent and the ability of patients to use the VR equipment.

The within-subject design with graded exposure to the VR intervention was chosen to reduce bias within the study, as it reduces the effect of patient-related confounding factors, such as biopsychosocial influences. Unfortunately this design may have consequently reduced the feasibility of accruing participants.

Each patient has to receive at least three inpatient dressing changes not requiring a general anaesthetic or ketamine. Those patients who are otherwise well enough to be discharged home return to outpatient clinic for dressing changes, so many only undergo a one or two study-appropriate dressing change following skin grafting and prior to discharge. For logistical reasons, the study is not currently being run in the outpatient clinic. A relatively high proportion of burn patients with longer lengths of stay have self-harmed (mean 22 days). As many as 82 % suffer from mental illness [33], with many excluded from the study due to the inability to provide informed consent or an unwillingness to take part. In summary, those most suitable for enrolment in the trial are usually those who are discharged the earliest so are unable to complete all three stages of data collection. In order to improve the recruitment rate, future trial designers could reduce the number of required dressing changes to two. This would allow a within-subject comparison of control conditions and either passive or active VR for each patient, albeit with a larger sample size. The research team should be aware that prison inmates can be enrolled, as the study does not continue when the patient is returned to prison.

The majority of surviving patients with a longer length of stay are the older patients (mean 23 days), those with medical comorbidities and those with large burns [33–35]. Whilst increasing age is associated with higher mortality for a given percentage of burn, this rate is improving and there are increasing numbers of older survivors of burns [34]. Over half of the over 65s admitted to the burn unit are treated conservatively [35]. All these patients are screened, and many undergo the prerequisite number of appropriate dressing changes; however, many have been found to be too frail to attempt to use the VR system. This finding needs to be explored further. Future studies should include a thorough patient capabilities and ergonomics assessment, as part of the human-centred design process [22], prior to undertaking clinical trials.

## Trial status

At the time of submission of this protocol (10 February 2015), enrolment into the study was ongoing and eight patients had been enrolled.

## Abbreviations

ANOVA: Analysis of variance
COTS: Commercial off the shelf
fMRI: functional magnetic resonance imaging
NRS: Numerical rating scale
QEHB: Queen Elizabeth Hospital Birmingham
VR: Virtual reality
VRET: Virtual restorative environment therapy

## Footnotes

1 Immersion is the term used to describe the amount of stimulation imposed by the VR system on the body’s sensory inputs. Increased immersion can be achieved, for example, by use of haptic feedback systems. Presence is the term used to describe the psychological consequence of technological immersion; the sensation of ‘being there’ within the virtual environment.
